# Prognostic Value of BIRC5 in Lung Adenocarcinoma Lacking EGFR, KRAS, and ALK Mutations by Integrated Bioinformatics Analysis

**DOI:** 10.1155/2019/5451290

**Published:** 2019-04-09

**Authors:** Yajuan Cao, Weikang Zhu, Wanqing Chen, Jianchun Wu, Guozhen Hou, Yan Li

**Affiliations:** Shanghai Municipal Hospital of Traditional Chinese Medicine, Shanghai University of Traditional Chinese Medicine, Shanghai 200071, China

## Abstract

**Objective:**

This study was aimed at investigating the prognostic significance of Baculoviral IAP repeat containing 5 (BIRC5) in lung adenocarcinoma (LAD) lacking EGFR, KRAS, and ALK mutations (triple-negative (TN) adenocarcinomas).

**Methods:**

The gene expression profiles were obtained from Gene Expression Omnibus (GEO). The identification of the differentially expressed genes (DEGs) was performed by GeneSpring GX. Gene set enrichment analysis (GSEA) was used to execute gene ontology function and pathway enrichment analysis. The protein interaction network was constructed by Cytoscape. The hub genes were extracted by MCODE and cytoHubba plugin from the network. Then, using BIRC5 as a candidate, the prognostic value in LAD and TN adenocarcinomas was verified by the Kaplan-Meier plotter and The Cancer Genome Atlas (TCGA) database, respectively. Finally, the mechanism of BIRC5 was predicted by a coexpressed network and enrichment analysis.

**Results:**

A total of 38 upregulated genes and 121 downregulated genes were identified. 9 hub genes were extracted. Among them, the mRNA expression of 5 genes, namely, BIRC5, MCM4, CDC20, KIAA0101, and TRIP13, were significantly upregulated among TN adenocarcinomas (all *P* < 0.05). Notably, only the overexpression of BIRC5 was associated with unfavorable overall survival (OS) in TN adenocarcinomas (log rank *P* = 0.0037). TN adenocarcinoma patients in the BIRC5 high-expression group suffered from a significantly high risk of distant metastasis (*P* = 0.046), advanced N stage (*P* = 0.033), and tumor-bearing (*P* = 0.031) and deceased status (*P* = 0.003). The mechanism of BIRC5 and coexpressed genes may be linked closely with the cell cycle.

**Conclusion:**

Overexpressed in tumors, BIRC5 is associated with unfavorable overall survival in TN adenocarcinomas. BIRC5 is a potential predictor and therapeutic target in TN adenocarcinomas.

## 1. Introduction

Lung adenocarcinoma (LAD), which accounts for more than 50% of all lung cancers, is the most frequent pathological type [1]. There have been several studies focusing on gene mutations in LAD. Epidermal growth factor receptor (EGFR) mutation occurs in up to 20–50% of LAD cases in Asian countries [2], making patients respond to EGFR tyrosine kinase inhibitors (TKI), such as gefitinib; thus, they demonstrate modest survival benefits [3]. A previous report revealed that Kirsten rat sarcoma viral oncogene homolog (KRAS) gene mutations occur more frequently in patients from western countries (approximately 20-25%) than in those from Asian countries [4]. Unfortunately, KRAS inhibitors are still being evaluated in experiments. On the other hand, anaplastic lymphoma kinase (ALK) rearrangements occur in 3-5% of patients with non-small-cell lung cancer (NSCLC) [5], and ALK-positive patients can benefit from ALK inhibitors [6]. In contrast, a subset of LAD patients remain without EGFR, KRAS, and ALK mutations (triple-negative (TN) adenocarcinomas). A poor prognosis of TN adenocarcinomas is attributed to a lack of sufficient genetic information and therapeutic targets.

Gene expression profiling is a powerful method that can aid in identifying important mechanistic pathways. High-throughput bioinformatic approaches provide strong tools to comprehensively quantify gene expression [7]. Bioinformatic analysis, including mechanistic pathway and gene ontology strategies, enable investigators to assess cell states by categorizing discrete and recurrently upregulated or downregulated genes [8]. Currently, the acquisition of big data on multiple platforms has increasingly been performed not only to understand the mechanisms of oncogenesis inherent to specific cancers, but also to provide us with drug targets and molecular diagnostic and prognostic factors, as well as biomarkers for patient risk stratification and treatment [9–11].

In our analysis, we identified differentially expressed genes (DEGs) between tumor and nontumor tissues in LAD patients from the Gene Expression Omnibus (GEO) database, enriched potential pathways, and ontology functions using gene set enrichment analysis (GSEA) and conducted survival analysis of hub genes, in the hope of assessing the prognostic significance and potential molecular mechanism of prognostic candidates for TN adenocarcinoma patients.

## 2. Materials and Methods

### 2.1. Source of Data

Three mRNA expression datasets, namely, GSE10072, GSE32863, and GSE85841, were obtained from the GEO database (https://www.ncbi.nlm.nih.gov/geo/). GSE10072 was based on the GPL96 platform (Affymetrix Human Genome U133A Array), and it included 58 LAD and 49 nontumor tissues. GSE32863 was based on GPL6884 (Illumina HumanWG-6 v3.0 expression beadchip), and it included 58 LAD and 58 adjacent nontumor tissues. GSE85841 was based on GPL20115 (Agilent-067406 Human CBC lncRNA+mRNA microarray V4.0), and it included 8 LAD and 8 adjacent nontumor tissues. All of the data can be viewed online.

### 2.2. Differentially Expressed Gene (DEG) Identification

GeneSpring GX 12.5 software (Agilent Technologies Inc., Santa Clara, CA, US) was used to screen DEGs in the three datasets. The gene expression data was normalized by the robust multiarray average (RMA) quantile normalization analysis algorithm. Quality control was performed by 3D principal component analysis (PCA) scores. *t*-Test unpaired analysis and an asymptotic *P* value computation with Benjamini-Hochberg multiple testing correction was used to detect DEGs whose fold change (FC) ≥ 2 with a false discovery rate (FDR) cutoff < 0.05 between LAD and adjacent nontumor tissues.

### 2.3. Enrichment Analysis of DEG Candidates

An enrichment analysis was performed on pathways and gene ontology to determine the functions of the overlapping DEGs by using gene set enrichment analysis (GSEA) (version 3.0, http://software.broadinstitute.org/gsea/), a computational method that determines whether a defined set of genes shows statistical significance [12, 13].

### 2.4. Protein-Protein Interaction (PPI) Network Construction

The Search Tool for the Retrieval of Interacting Genes (STRING) database (version 10.5, https://string-db.org/) was used to analyze potential interactions between overlapping genes at the protein level, and a medium confidence score > 0.4 was considered significant. Subsequently, the PPI network was constructed by Cytoscape software (version 3.6.0, https://cytoscape.org/). The Molecular Complex Detection (MCODE) plugin was used for searching the most significant module from the network [14]. The MCODE criteria for selection were as follows: MCODE scores ≥ 5, degree cutoff = 2, node score cutoff = 0.2, K − core = 2, and max depth = 100.

### 2.5. Hub Node Extraction and Verification

We used the cytoHubba plugin, which integrates eleven topological analysis methods and six centralities with the Maximal Clique Centrality (MCC) algorithm, to explore the top 10 candidates as hub genes in the PPI network [15]. Finally, we combined the results of MCODE and cytoHubba analysis, and candidates were extracted from the network. UCSC Xena (version 2.0, https://xena.ucsc.edu/welcome-to-ucsc-xena/), a securely analyzed and visualized functional genomic dataset in TCGA datasets, was utilized to verify the differential expression of these candidates between LAD and nontumor tissues.

### 2.6. Survival Analysis

We investigated the prognostic significance of hub genes among TN adenocarcinomas. Clinical data, mRNA expression, and mutation data in the lung adenocarcinoma (TCGA, Provisional) database were obtained from the cBioPortal online platform (version 1.17.1, http://www.cbioportal.org/) [16, 17]. TN adenocarcinoma patients were included for further analysis to investigate the association between BIRC5 and survival by using the median cutoff of mRNA expression. The Kaplan-Meier plotter (http://kmplot.com), an online database which includes both clinical and expression data, was utilized to verify the relationship between BIRC5 and the survival time of LAD [18].

### 2.7. Coexpression Network Construction and Topology Analysis

To further explore the mechanism of BIRC5 as a potential prognostic significance molecule for TN adenocarcinomas, BIRC5 and coexpression genes were analyzed by using cBioPortal. The coexpression network was visualized by Cytoscape, and degree ≥ 160 (average degree) was set as a cutoff criterion.

### 2.8. Statistical Analysis

The differences of gene expression between the individual groups were analyzed using the Student *t*-test, Mann–Whitney *U* test, chi-squared test, and Ridit analysis based on the types of variables. Kaplan-Meier analysis was conducted by GraphPad Prism 7 (GraphPad Software Inc., CA, USA). Factors associated with LAD overall survival were assessed by both Cox univariate and multivariate analyses. Only covariates significantly associated with outcomes in the univariate analysis (two-sided *P* < 0.10) were included in the multivariate model. PASW Statistics software version 23.0 from SPSS Inc. (Chicago, IL, USA) was used. A two-tailed *P* < 0.05 was considered significant for all tests.

## 3. Results

### 3.1. Identification of DEGs

**Figure 1:**
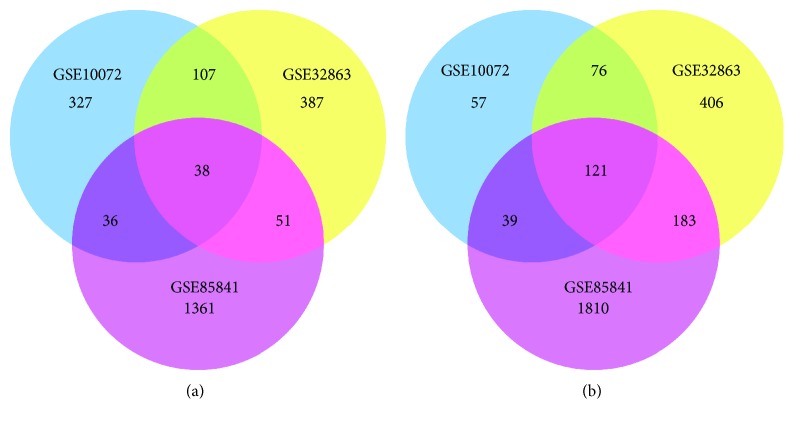
DEGs between tumor and adjacent tissues in GSE10072, GSE32863, and GSE85841. (a) Venn diagram of 38 overlapping upregulated genes. (b) Venn diagram of 121 overlapping downregulated genes.

A total of 508, 583, and 1486 upregulated DEGs and 293, 787, and 2153 downregulated DEGs were identified in GSE10072, GSE32863, and GSE85841 profiles, respectively. Using a Venn diagram, 159 overlapping genes, including 38 upregulated genes (Figure 1(a)) and 121 downregulated ones (Figure 1(b)), were identified among these three GEO profiles.

### 3.2. Functional Enrichment Analysis of DEGs

**Figure 2:**
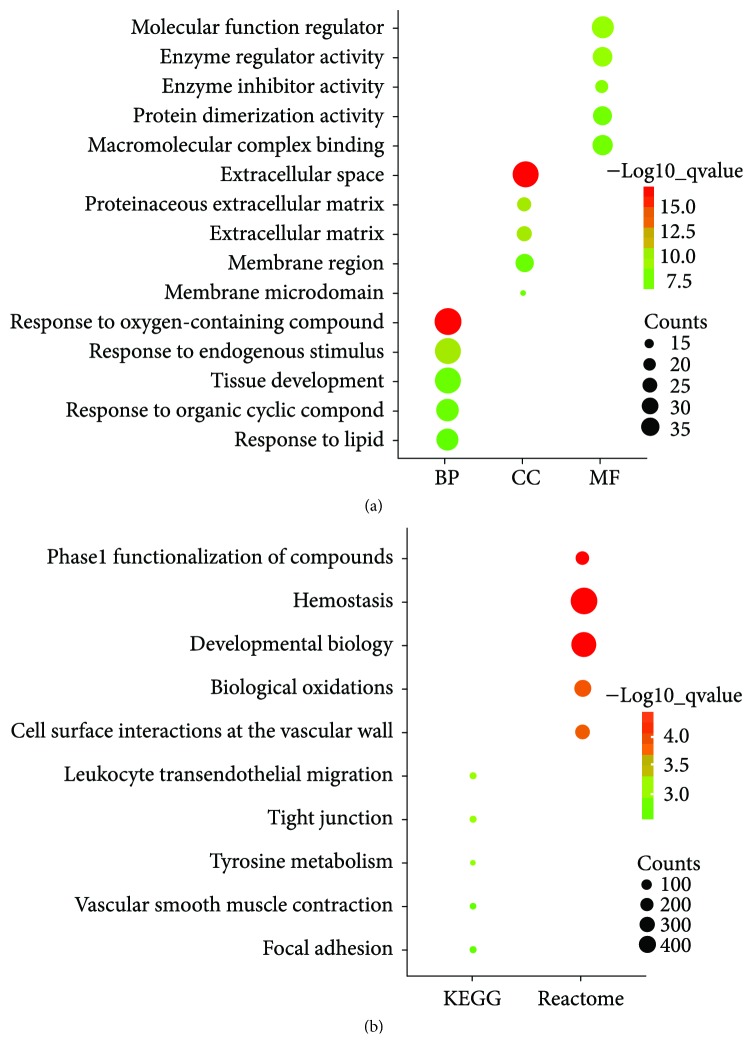
Enrichment analysis of DEGs. (a) GO analysis: top 5 of biological processes (BP), cellular components (CC), and molecular function (MF). (b) Pathway analysis: top 5 KEGG pathways and Reactome pathways.

To classify the biological function of DEGs, gene ontology and pathway enrichment analyses were performed using GSEA. As shown in Figure 2(a), GO analysis revealed that 159 DEGs were significantly enriched in the following biological processes (BP), including response to an oxygen containing compound, response to an endogenous stimulus, tissue development, response to an organic cyclic compound, and response to lipids. Cellular component (CC) analysis showed that extracellular space, proteinaceous extracellular matrix, extracellular matrix, membrane microdomain, and membrane region were mostly classifications. In terms of molecular functions (MF), the DEGs were mainly associated with the molecular function regulator, enzyme regulator activity, enzyme inhibitor activity, protein dimerization activity, and macromolecular complex binding. In addition, the pathways that had the most significant enrichment terms were shown by KEGG and Reactome. As shown in Figure 2(b), KEGG pathways included leukocyte transendothelial migration, tight junction, tyrosine metabolism, vascular smooth muscle contraction, and focal adhesion and Reactome pathways included hemostasis, developmental biology, phase 1 functionalization of compounds, biological oxidations, and cell surface interactions at the vascular wall.

### 3.3. PPI Network Construction and Hub Gene Identification

**Figure 3:**
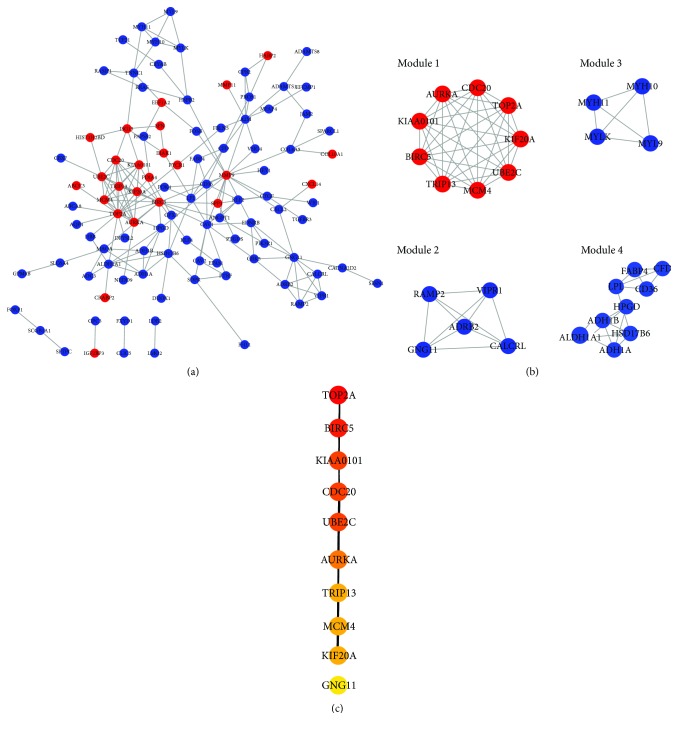
PPI network construction. (a) PPI network: the red nodes represent upregulated DEGs and the blue nodes represent downregulated DEGs. (b) 4 modules were extracted by MCODE. (c) The top 10 hub genes were chosen by cytoHubba, and the more forward ranking is represented by a redder color.

Protein interactions among DEGs were predicted by the STRING online platform. The PPI network, which included a total of 104 nodes and 188 edges, was visualized by Cytoscape (Figure 3(a)). Subsequently, we utilized the MCODE plugin to analyze the whole network and 4 modules were chosen (Figure 3(b); Module 1, Module 2, Module 3, and Module 4). Among them, Module 1 showed the most significant scores.

To further identify the hub genes in the PPI network, we used the cytoHubba plugin to extract the following top 10 genes as hub genes (Figure 3(c)): TOP2A, BIRC5, KIAA0101, CDC20, UBE2C, AURKA, TRIP13, MCM4, KIF20, and GNG11. Notably, 9 genes of Module 1 were all included in the hub genes. Finally, we confirmed 9 candidates for our further study: TOP2A, BIRC5, KIAA0101, CDC20, UBE2C, AURKA, TRIP13, MCM4, and KIF20. Interestingly, all of candidates were upregulated in LAD tissues.

### 3.4. Differentially Expressed Analysis of Hub Genes

**Figure 4:**
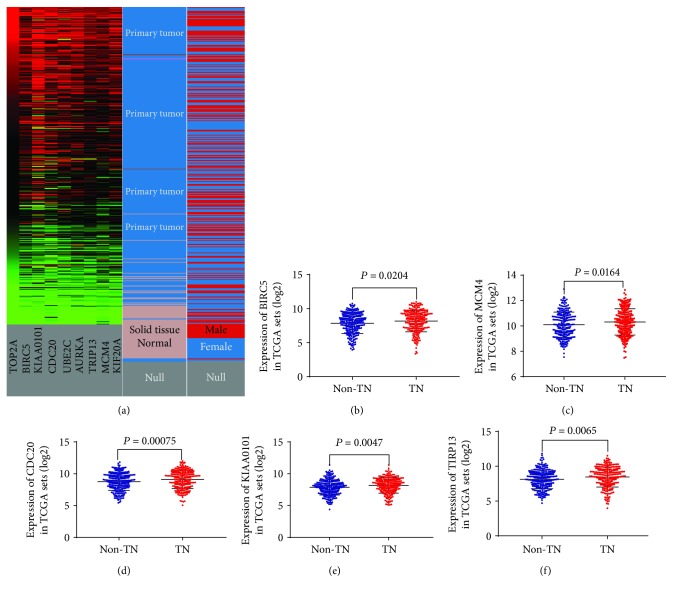
mRNA expression of hub genes. (a) The mRNA expression of 9 hub genes (TOP2A, BIRC5, KIAA0101, CDC20, UBE2C, AURKA, TRIP13, MCM4, and KIF20) between LAD and normal tissues based on the UCSC Xena database. 5 hub genes, namely, BIRC5 (b), MCM4 (c), CDC20 (d), KIAA0101 (e), and TIRP13 (f), were more significantly upregulated in triple-negative (TN) lung adenocarcinomas based on TCGA database than in non-triple-negative (non-TN) lung adenocarcinomas.

To verify the results of the GEO dataset analysis, UCSC Xena was carried out to exhibit the differential expression of 9 hub genes between LAD and nontumor tissues. As shown in Figure 4(a), the hierarchical clustering revealed similar results in the TCGA datasets and all of 9 hub genes were more upregulated in LAD than in nontumor tissues. Following which, to investigate the relationship of hub genes and TN adenocarcinomas, the mRNA expression levels of the 9 hub genes were calculated between TN adenocarcinomas and non-TN adenocarcinomas in TCGA datasets. The profiles of a total of 544 LAD patients were available for analysis. As shown briefly in Figures 4(b)–4(f), the mRNA expression levels of 5 genes, namely, BIRC5, MCM4, CDC20, KIAA0101, and TRIP13, were significantly upregulated in TN adenocarcinomas (all*P* < 0.05) compared with those in non-TN adenocarcinomas, while the mRNA expression levels of 4 genes, namely, TOP2A (*P* = 0.259), UBE2C (*P* = 0.0569), AURKA (*P* = 0.0778), and KIF20 (*P* = 0.1634) were not upregulated in TN adenocarcinomas.

### 3.5. Prognostic Analysis of BIRC5 in TN Adenocarcinomas

**Figure 5:**
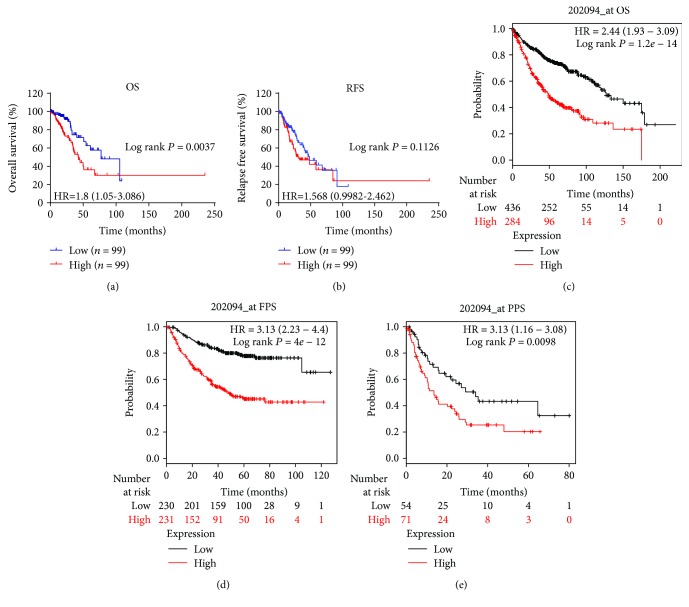
Associations between BIRC5 and survival times. OS (a) and RFS (b) in triple-negative (TN) lung adenocarcinomas based on TCGA database. OS (c), FPS (d), and PPS (e) in lung adenocarcinomas (LAD) based on the Kaplan-Meier plotter database. The red lines represent patients with high BIRC5 expression, and the blue and black lines represent patients with a low BIRC5 expression.

The TCGA mRNA profile and clinical and mutation datasets were matched, and 199 TN adenocarcinomas were grouped by the median cutoff of the mRNA expression levels of 5 genes. Notably, only the high level of mRNA expression of BIRC5 was associated with poor overall survival (OS, log rank *P* = 0.0037, Figure 5(a)), but not the relapse-free survival (RFS, log rank *P* = 0.1126, Figure 5(b)). Meanwhile, this significant difference was not observed in MCM4, KIAA0101, CDC20, and TRIP13 among TN adenocarcinomas.

We described clinicopathological features in the BIRC5 high- and low-expression groups in TN adenocarcinomas, as shown in Table 1. The BIRC5 high-expression group had more male patients (*P* = 0.023) and smoked packs per year (*P* = 0.025), and TN adenocarcinoma patients in the BIRC5 high-expression group suffered from a significantly higher risk of distant metastasis (*P* = 0.046) and advanced N stage (*P* = 0.033). Tumor-bearing (*P* = 0.031) and deceased status (*P* = 0.003) are also related with BIRC5 high expression. There were also more patients who received additional radiation therapy in the BIRC5 high-expression group than those in the BIRC5 low-expression group (26.3% vs. 14.1%, *P* = 0.034, Table 1).

In order to further evaluate the prognostic significance of BIRC5 in TN adenocarcinomas, the potential risk factors associated with OS of TN adenocarcinoma patients were analyzed using Cox regression analysis. Univariate analysis revealed that high-level BIRC5, recurrence/metastasis, new tumor event after initial treatment, advanced TNM stage, and person neoplasm cancer status with tumor are potential risk factors significantly associated with OS in TN adenocarcinoma patients (all *P* < 0.05, Table 2). When these variables were included in the multivariate analysis using the forward LR method, high-level BIRC5, advanced TNM stage, and person neoplasm cancer status with tumor were the risk factors that significantly contributed to worse OS in TN adenocarcinoma patients (*P* = 0.015, *P* = 0.049, and *P* < 0.001, respectively, Table 2).

Additionally, the Kaplan-Meier plotter was utilized to verify if the high level of mRNA expression of BIRC5 predicted an unfavorable OS (log rank *P* = 1.2*e* − 14, Figure 5(c)), progression-free survival (FPS, log rank *P* = 0.0098, Figure 5(d)), and postprogression survival (PPS, log rank *P* = 0.0098, Figure 5(e)) among LAD.

### 3.6. Coexpression Network Construction and Enrichment Analysis of Coexpression Genes

**Figure 6:**
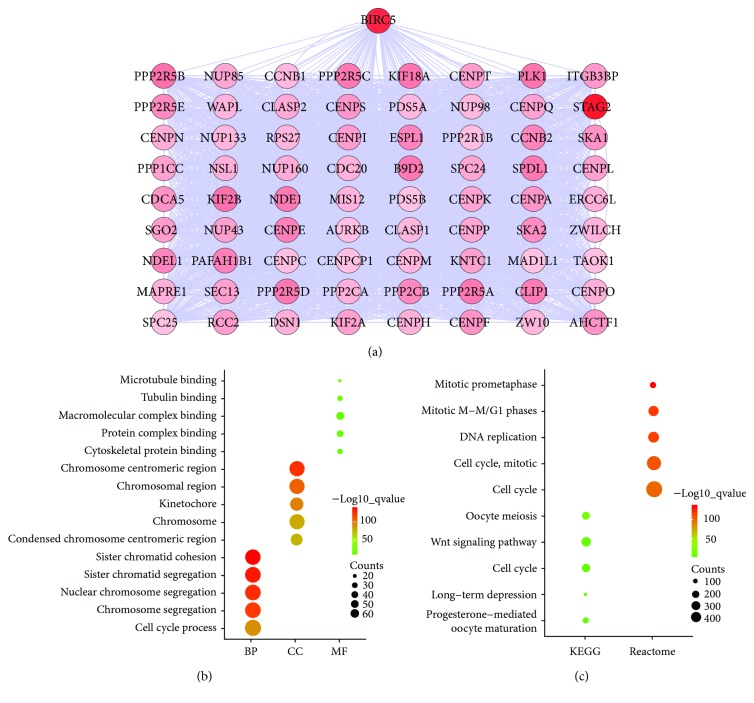
Coexpression network construction and enrichment analysis. (a) Coexpression network: the higher degree is represented by a redder color. (b) GO analysis: top 5 of biological processes (BP), cellular components (CC), and molecular function (MF). (c) Pathway analysis: top 5 KEGG pathways and Reactome pathways.

BIRC5 indicated a significant prognostic correlation, and genes coexpressed with BIRC5 were predicted using the cBioPortal online platform. As shown in Figure 6(a), the coexpression network was visualized by Cytoscape software, and the network included 73 nodes and 2,613 edges. According to the topological analysis of the coexpression network, STAG2 is the most relevant gene with BIRC5.

We next imported BIRC5 and the coexpressed genes into GSEA to carry out enrichment analysis. As shown in Figure 6(b), GO terms were most significantly enriched in BP, including sister chromatid cohesion, sister chromatid segregation, nuclear chromosome segregation, chromosome segregation, and cell cycle process. CC was involved in the chromosome centromeric region, chromosomal region, kinetochore, chromosome, and condensed chromosome centromeric region. MF referred to microtubule binding, tubulin binding, macromolecular complex binding, protein complex binding, and cytoskeletal protein binding. Pathway enrichment analysis in Figure 6(c) showed that oocyte meiosis, wnt signaling pathway, cell cycle, long-term depression, and progesterone-mediated oocyte maturation were enriched in the KEGG pathway. Mitotic prometaphase; mitotic M-M/G1 phases; DNA replication; cell cycle, mitotic; and cell cycle were gathered in the Reactome pathway. Briefly, enrichment results suggested that BIRC5 and coexpressed genes may be linked closely with the cell cycle.

## 4. Discussion

BIRC5, 14.7 kb long, is a mitotic spindle checkpoint gene, located near the telomeric end of chromosome 17 [19]. BIRC5 is not only an essential protein molecule for the regulation of mitosis and apoptosis, but it is also involved in pathological processes [20]. It has been shown that BIRC5 was upregulated in tumor tissues. In a variety of human cancers, such as breast cancer [21], pancreatic cancer [22], hepatocarcinoma [23], neuroblastoma [24], and esophageal carcinoma [25], high expression of BIRC5 indicated poor clinical outcomes. In addition, circulating IgG antibodies derived from BIRC5 could serve as a biomarker for malignant glial tumor [26] and early diagnosis of cervical cancer [27]. Therefore, BIRC5, as a target, might be useful for the treatment of drugs in gastric and colorectal cancers [28]. Targeting BIRC5 may be a promising strategy against esophageal tumor relapse and chemoradioresistance [29].

In this study, we identified that the high level of mRNA expression of BIRC5 not only was related to unfavorable outcomes of LAD, but it also indicated a shorter OS of TN adenocarcinomas. Coexpression and enrichment analysis revealed that BIRC5 may serve as a critically coexpressed gene for mediating the cell cycle-related signaling pathway. We assumed that the oncogenic role and prognostic value of BIRC5 in TN adenocarcinomas can be ascribed to this “cell cycle-mediated” mechanism.

We therefore summarize previous publications. First of all, BIRC5 was overexpressed in LAD compared to normal controls [30–32]. Our findings are consistent with existing investigations. Secondly, the potential prognostic and therapeutic value of BIRC5 in LAD was evaluated. Plasma anti-BIRC5 IgG may be a useful marker for the assessment of prognosis of NSCLC [33]. BIRC5 expression is an independent poor prognostic marker in LAD [34]. In the aspect of regulating the sensitivity of LAD cells to anticancer drugs, BIRC5 also plays a critical role [35]. In terms of EGFR or RAS mutant NSCLC treatment, BIRC5 contributes to induced cytotoxicity [36]. Briefly, BIRC5 may constitute a valuable biomarker and therapeutic target. Notably, compared with the previous investigations, our study provided new evidence that BIRC5 plays an important role in the prognosis of TN adenocarcinomas. Finally, the mechanism behind the effect of BIRC5 on tumors was described. Survivin, encoded by BIRC5, has a bifunctional molecule effect on apoptosis and cell proliferation, mainly from the two phosphorylation sites on its different domains (Thr34 and Thr117). Thr117 is a key site on the regulation of proliferation and cell cycle, and Thr34 is involved in cell apoptosis [37]. BIRC5 has been identified as a critical target involved in a variety of cancer cell signaling pathways, and the upregulation of BIRC5 may serve as a role in the following mechanisms: critically antagonizing caspase-dependent apoptosis and activating P53 and its downstream target P21, which stalls cell cycle progression as a cyclin-dependent kinase inhibitor (CDKi) [38]. Additionally, BIRC5 promotes the migration and invasion of tumor cells mediated by the TGF-*β* pathway [39] and the PI3K/AKT pathway [25], interacts with proteins associated with DNA damage repair [40], and regulates tumor cell proliferation mediated via the *β*-catenin pathway [41]. These facts explained why BIRC5 was mainly involved in the regulation of cell cycle and apoptosis; meanwhile, it is supported in our study that the overexpression of BIRC5 promoted the development of TN adenocarcinomas, which may be via regulating the cell cycle signaling pathway.

In conclusion, for TN adenocarcinomas, BIRC5 may serve as a promising prognostic predictor and therapeutic target. The upregulation of BIRC5 may enable the activation of the cell cycle signaling pathway to participate in the development of this specific type of LAD. Our findings provide a novel insight into TN adenocarcinomas. However, cancer is a result of highly complex molecular mechanisms, and more experimental studies are necessary to clarify the cell cycle-related signaling for TN adenocarcinomas in the context of BIRC5.

## Figures and Tables

**Table 1:** Baseline characteristics of TN adenocarcinoma patients between the BIRC5 high- and low-expression groups.

Variables	BIRC5 expression level		*P* value
	Low (*n* = 99)	High (*n* = 99)	
Age, mean ± SD (years)	67 ± 8	63 ± 11	**0.045**
Male, *n* (%)	40 (40.4)	56 (56.6)	**0.023**
Overall survival, median (IQR) (days)	704.5 (745)	658 (579)	0.427
Recurrence-free survival, median (IQR) (days)	624.5 (701)	536 (641)	0.439
Additional pharmaceutical therapy, *n* (%)	12 (12.1)	15 (15.2)	0.534
Additional radiation therapy, *n* (%)	14 (14.1)	26 (26.3)	**0.034**
Targeted molecular therapy, *n* (%)	26 (26.3)	31 (31.3)	0.433
Partial response, *n* (%)	1 (1.0)	2 (2.0)	0.561
Stable disease, *n* (%)	5 (5.1)	7 (7.1)	0.551
Progressive disease, *n* (%)	20 (20.2)	30 (30.3)	0.096
New primary tumor, *n* (%)	6 (6.1)	1 (1.0)	0.053
Distant metastasis, *n* (%)	10 (10.1)	20 (20.2)	**0.046**
Locoregional recurrence, *n* (%)	13 (13.1)	6 (6.1)	0.091
New tumor event after initial treatment, *n* (%)	33 (33.3)	44 (44.4)	0.109
Smoked packs per year, median (IQR), *n*	35 (25.5)	44 (22)	**0.025**
TNM stage, *n* (%)			0.194
I	64 (64.6)	52 (52.5)	
II	22 (22.2)	26 (26.3)	
III	9 (9.1)	18 (18.2)	
IV	4 (4.0)	3 (3.0)	
T stage, *n* (%)			0.112
T1	44 (44.4)	33 (33.3)	
T2	44 (44.4)	58 (58.6)	
T3	10 (10.1)	6 (6.1)	
T4	0 (0)	2 (2.0)	
Tx	1 (1.0)	0 (0)	
N stage, *n* (%)			**0.033**
N0	72 (72.7)	59 (60.0)	
N1	15 (15.2)	24 (24.2)	
N2	8 (8.1)	15 (15.2)	
N3	0 (0)	1 (1.0)	
Nx	4 (4.0)	0 (0)	
M stage, *n* (%)			0.645
M0	62 (62.6)	68 (68.7)	
M1	3 (3.0)	3 (3.0)	
Mx	33 (33.3)	27 (27.3)	
Person neoplasm cancer status, *n* (%)			**0.031**
With tumor	28 (28.3)	42 (42.4)	
Tumor-free	68 (68.7)	53 (53.5)	
Tobacco smoking history indicator, *n* (%)			0.630
Current smoker	9 (9.1)	12 (12.1)	
Current reformed smoker for ≤15 years	17 (17.2)	12 (12.1)	
Current reformed smoker for >15 years	12 (12.1)	11 (11.1)	
Lifelong nonsmoker	7 (7.1)	4 (4.0)	
Vital status, *n* (%)			
Living	79 (80.0)	60 (60.6)	**0.003**
Deceased	20 (20.2)	39 (39.4)	

IQR, interquartile range.

**Table 2:** Cox regression analysis of risk factors associated with overall survival of TN adenocarcinoma patients.

Covariates	Univariate analysis, HR (95% CI)	*P* value	Multivariate analysis, HR (95% CI)	*P* value
BIRC5, high vs. low	2.184 (1.271-3.753)	**0.005**	2.029 (1.149-3.581)	**0.015**
Additional pharmaceutical therapy, yes vs. no	1.443 (0.773-2.693)	0.249		
Targeted molecular therapy, yes vs. no	0.768 (0.438-1.345)	0.456		
Age, per increase of 1 year	1.013 (0.985-1.041)	0.375		
Gender, male vs. female	1.064 (0.635-1.781)	0.814		
Recurrence/metastasis, yes vs. no	1.890 (1.127-3.171)	**0.016**		
New tumor event after initial treatment, yes vs. no	3.936 (2.215-6.994)	**<0.001**		
TNM staging, per increase of 1 stage	1.588 (1.231-2.048)	**<0.001**	1.300 (1.001-1.687)	**0.049**
Person neoplasm cancer status, with tumor vs. tumor-free	9.554 (4.810-18.975)	**<0.001**	8.539 (4.259-17.120)	**<0.001**
Tobacco smoking history, yes vs. no	0.739 (0.299-1.825)	0.512		

## Data Availability

The data used to support the findings of this study are available from the corresponding author upon request.

## References

[1] Bray F., Ferlay J., Soerjomataram I., Siegel R. L., Torre L. A., Jemal A. (2018). Global cancer statistics 2018: GLOBOCAN estimates of incidence and mortality worldwide for 36 cancers in 185 countries. *CA: A Cancer Journal for Clinicians*.

[2] Tokumo M., Toyooka S., Kiura K. (2005). The relationship between epidermal growth factor receptor mutations and clinicopathologic features in non-small cell lung cancers. *Clinical Cancer Research*.

[3] Thatcher N., Chang A., Parikh P. (2005). Gefitinib plus best supportive care in previously treated patients with refractory advanced non-small-cell lung cancer: results from a randomised, placebo-controlled, multicentre study (Iressa Survival Evaluation in Lung Cancer). *The Lancet*.

[4] Dogan S., Shen R., Ang D. C. (2012). Molecular epidemiology of *EGFR* and *KRAS* mutations in 3,026 lung adenocarcinomas: higher susceptibility of women to smoking-related *KRAS*-mutant cancers. *Clinical Cancer Research*.

[5] Gainor J. F., Varghese A. M., Ou S. H. I. (2013). *ALK* rearrangements are mutually exclusive with mutations in *EGFR* or *KRAS*: an analysis of 1,683 patients with non-small cell lung cancer. *Clinical Cancer Research*.

[6] Camidge D. R., Kim H. R., Ahn M. J. (2018). Brigatinib versus crizotinib in *ALK*-positive non-small-cell lung cancer. *The New England Journal of Medicine*.

[7] Leipzig J. (2017). A review of bioinformatic pipeline frameworks. *Briefings in Bioinformatics*.

[8] Kawakami A., Fisher D. E. (2016). Bioinformatic analysis of gene expression for melanoma treatment. *Journal of Investigative Dermatology*.

[9] Le Large T. Y. S., Mato Prado M., Krell J. (2016). Bioinformatic analysis reveals pancreatic cancer molecular subtypes specific to the tumor and the microenvironment. *Expert Review of Molecular Diagnostics*.

[10] Bennani-Baiti N., Bennani-Baiti I. M. (2015). Cancer bioinformatic methods to infer meaningful data from small-size cohorts. *Cancer Informatics*.

[11] Zhou X., Du Y. L., Jin P., Ma F. (2015). Bioinformatic analysis of cancer-related microRNAs and their target genes. *Yi Chuan*.

[12] Subramanian A., Tamayo P., Mootha V. K. (2005). Gene set enrichment analysis: a knowledge-based approach for interpreting genome-wide expression profiles. *Proceedings of the National Academy of Sciences of the United States of America*.

[13] Mootha V. K., Lindgren C. M., Eriksson K. F. (2003). PGC-1*α*-responsive genes involved in oxidative phosphorylation are coordinately downregulated in human diabetes. *Nature Genetics*.

[14] Anitha P., Anbarasu A., Ramaiah S. (2016). Gene network analysis reveals the association of important functional partners involved in antibiotic resistance: a report on an important pathogenic bacterium *Staphylococcus aureus*. *Gene*.

[15] Chin C. H., Chen S. H., Wu H. H., Ho C. W., Ko M. T., Lin C. Y. (2014). cytoHubba: identifying hub objects and sub-networks from complex interactome. *BMC Systems Biology*.

[16] Gao J., Aksoy B. A., Dogrusoz U. (2013). Integrative analysis of complex cancer genomics and clinical profiles using the cBioPortal. *Science Signaling*.

[17] Cerami E., Gao J., Dogrusoz U. (2012). The cBio cancer genomics portal: an open platform for exploring multidimensional cancer genomics data. *Cancer Discovery*.

[18] Gyorffy B., Surowiak P., Budczies J., Lanczky A. (2013). Online survival analysis software to assess the prognostic value of biomarkers using transcriptomic data in non-small-cell lung cancer. *PLoS One*.

[19] Wheatley S., Mcneish I. (2005). Survivin: a protein with dual roles in mitosis and apoptosis. *International Review of Cytology*.

[20] Altieri D. C. (2003). Survivin, versatile modulation of cell division and apoptosis in cancer. *Oncogene*.

[21] Hamy A. S., Bieche I., Lehmann-Che J. (2016). *BIRC5* (survivin): a pejorative prognostic marker in stage II/III breast cancer with no response to neoadjuvant chemotherapy. *Breast Cancer Research and Treatment*.

[22] Zhou L., Lu J., Liang Z. Y. (2018). High nuclear Survivin expression as a poor prognostic marker in pancreatic ductal adenocarcinoma. *Journal of Surgical Oncology*.

[23] Tian Q. G., Wu Y. T., Liu Y. (2018). Expressions and correlation analysis of HIF-1*α*, survivin and VEGF in patients with hepatocarcinoma. *European Review for Medical and Pharmacological Sciences*.

[24] Hagenbuchner J., Kiechl-Kohlendorfer U., Obexer P., Ausserlechner M. J. (2016). BIRC5/Survivin as a target for glycolysis inhibition in high-stage neuroblastoma. *Oncogene*.

[25] Shang X., Liu G., Zhang Y. (2018). Downregulation of BIRC5 inhibits the migration and invasion of esophageal cancer cells by interacting with the PI3K/Akt signaling pathway. *Oncology Letters*.

[26] Kafadar D., Yaylim I., Kafadar A. M. (2018). Investigation of *survivin* gene polymorphism and serum survivin levels in patients with brain tumors. *Anticancer Research*.

[27] Xu Y., Jin Y., Liu L., Zhang X., Chen Y., Wei J. (2015). Study of circulating IgG antibodies to peptide antigens derived from BIRC5 and MYC in cervical cancer. *FEBS Open Bio*.

[28] Lim T., Lee I., Kim J., Kang W. K. (2015). Synergistic effect of simvastatin plus radiation in gastric cancer and colorectal cancer: implications of BIRC5 and connective tissue growth factor. *International Journal of Radiation Oncology, Biology, Physics*.

[29] Zhou C., Zhang L., Xu P. (2018). Growth inhibition and chemo‑radiosensitization of esophageal squamous cell carcinoma by survivin‑shRNA lentivirus transfection. *Oncology Letters*.

[30] Duan L., Hu X., Jin Y., Liu R., You Q. (2016). Survivin protein expression is involved in the progression of non-small cell lung cancer in Asians: a meta-analysis. *BMC Cancer*.

[31] Hu S., Qu Y., Xu X., Xu Q., Geng J., Xu J. (2013). Nuclear survivin and its relationship to DNA damage repair genes in non-small cell lung cancer investigated using tissue array. *PLoS One*.

[32] Kapellos G., Polonifi K., Farmakis D. (2013). Overexpression of survivin levels in circulation and tissue samples of lung cancer patients. *Anticancer Research*.

[33] Zhao H., Zhang X., Han Z., Wang Z., Wang Y. (2018). Plasma anti-BIRC5 IgG may be a useful marker for evaluating the prognosis of nonsmall cell lung cancer. *FEBS Open Bio*.

[34] Sun P. L., Jin Y., Kim H. (2013). Survivin expression is an independent poor prognostic marker in lung adenocarcinoma but not in squamous cell carcinoma. *Virchows Archiv*.

[35] Zhou C., Zhu Y., Lu B., Zhao W., Zhao X. (2018). Survivin expression modulates the sensitivity of A549 lung cancer cells resistance to vincristine. *Oncology Letters*.

[36] Yan X., Li P., Zhan Y. (2018). Dihydroartemisinin suppresses STAT3 signaling and Mcl-1 and Survivin expression to potentiate ABT-263-induced apoptosis in non-small cell lung cancer cells harboring EGFR or RAS mutation. *Biochemical Pharmacology*.

[37] Wang L., Kang Y., Zheng W., Li L., Shi L., Ma X. (2014). Effect on apoptosis and cell cycle of recombinant double negative dominant mutation Survivin (T34/117A) in breast cancer cell B-Cap-37. *Biomedicine & Pharmacotherapy*.

[38] Rauch A., Carlstedt A., Emmerich C. (2018). Survivin antagonizes chemotherapy-induced cell death of colorectal cancer cells. *Oncotarget*.

[39] Wang B., Li X., Zhao G. (2018). miR-203 inhibits ovarian tumor metastasis by targeting BIRC5 and attenuating the TGF*β* pathway. *Journal of Experimental & Clinical Cancer Research*.

[40] Wani T. H., Surendran S., Mishra V. S., Chaturvedi J., Chowdhury G., Chakrabarty A. (2018). Adaptation to chronic exposure to sepantronium bromide (YM155), a prototypical survivin suppressant is due to persistent DNA damage-response in breast cancer cells. *Oncotarget*.

[41] Yang C. T., Li J. M., Li L. F., Ko Y. S., Chen J. T. (2018). Stomatin-like protein 2 regulates survivin expression in non-small cell lung cancer cells through *β*-catenin signaling pathway. *Cell Death & Disease*.

